# Immunomodulatory Effect of Pregnancy on Leukocyte Populations in Patients With Multiple Sclerosis: A Comparison of Peripheral Blood and Decidual Placental Tissue

**DOI:** 10.3389/fimmu.2019.01935

**Published:** 2019-08-16

**Authors:** Michela Spadaro, Serena Martire, Luca Marozio, Daniela Mastromauro, Elena Montanari, Simona Perga, Francesca Montarolo, Federica Brescia, Alessia Balbo, Giovanni Botta, Chiara Benedetto, Antonio Bertolotto

**Affiliations:** ^1^Clinical Neurobiology Unit, Neuroscience Institute Cavalieri Ottolenghi, Turin, Italy; ^2^Neurologia—CRESM (Centro Riferimento Regionale Sclerosi Multipla), Azienda Ospedaliera-Universitaria San Luigi Gonzaga, Turin, Italy; ^3^Department of Surgical Sciences, Obstetrics and Gynecology, University of Torino, Turin, Italy; ^4^Department of Neuroscience “Rita Levi Montalcini”, University of Turin, Turin, Italy; ^5^Department of Pathology, Città della Salute e della Scienza di Torino, Turin, Italy

**Keywords:** multiple sclerosis, pregnancy, immunomodulation, peripheral blood, placenta, regulatory T cells, CD56^bright^ NK cells, M2 monocytes

## Abstract

Pregnancy is a naturally occurring disease modifier of multiple sclerosis (MS) associated with a substantial reduction in relapse rate. To date, attempts to explain this phenomenon have focused on systemic maternal immune cell composition, with contradictory results. To address this matter, we compared the immunomodulatory effects of pregnancy on five leukocyte populations (i.e., CD4^+^ and CD8^+^ T cells, CD4^+^CD127^−^CD25^high^ regulatory T cells, CD56^bright^CD16^−^ NK cells, and CD14^+^CD163^+^ monocytes) in peripheral blood from different cohorts of MS patients and healthy women at different times of gestation, as well as in decidual samples from the placenta of MS patients and healthy women collected after delivery. For the first time to our knowledge, we observed that the frequency of these cell populations in the decidua is not different between MS patients and healthy women, suggesting that a physiological immune regulation may occur at the fetal-maternal interface. In peripheral blood, however, contrary to healthy women, in MS patients cell frequencies were not significantly altered by gestation. In particular, CD8^+^ T cells did not show differences between groups. CD4^+^ T cells were higher in non-pregnant MS compared to healthy women, while during pregnancy they remained constant in MS and increased in healthy women. Regulatory T cells were higher in non-pregnant controls compared to MS women, while the difference was reduced during gestation due to the decrease of regulatory T cell levels in healthy women. CD14^+^CD163^+^ monocytes did not show differences between groups. CD56^bright^CD16^−^ NK cells were not significantly different in non-pregnant MS compared to controls and increased in healthy women during gestation. In conclusion, our findings support the hypothesis that disease amelioration in MS patients during pregnancy may be due to a modulation of the immune cells functional activity rather than their frequency. Further studies exploring functional changes of these cells would be crucial to bring light into the complex mechanisms of pregnancy-induced tolerance and autoimmunity overall.

## Introduction

Multiple sclerosis (MS) is an autoimmune, inflammatory, and demyelinating disease of the central nervous system (CNS) (1). Although the etiopathogenesis is not completely understood, MS is thought to arise from the complex interplay of genetic causes and environmental triggers, which cause a breakdown of peripheral tolerance against CNS antigens (1, 2). This has been linked to defects in regulatory cells as well as resistance of autoreactive effector cells to suppression. MS probably begins with the activation of autoreactive CD4^+^ T helper type 1 (Th1) cells directed against CNS antigens in the periphery. After entering the CNS, the autoreactive T-cells may be reactivated by resident antigen presenting cells or by invading dendritic cells presenting the local CNS antigen(s). This reactivation triggers the recruitment and activation of additional cells to the areas of inflammation (e.g., B-cells, natural killer [NK] cells, myeloid cells), the secretion of various cytokines, chemokines, matrix metalloproteinases, and other mediators, and the activation of resident microglia and astrocytes, which finally results in the myelin damage (3).

Pregnancy is a naturally occurring disease modifier of MS associated with a 70% reduction in relapse rate in the third trimester. In contrast, the post-partum period entails increased relapse risk, which may be due to removal of protective factors related to pregnancy (4, 5). The principal effector of such an improvement is thought to be the immunomodulation of the maternal immune system induced by gestation (6, 7).

In physiological pregnancy, immunomodulation has been described to occur locally at the placental interface, through the direct interaction between trophoblastic (fetal) and decidual (maternal) cells, as well as peripherally in the maternal blood, leading to changes in both adaptive and innate immune responses (8, 9).

A better understanding of the mechanisms of fetal-maternal tolerance may provide insights into the biology of MS disease as well as inputs for the development of new treatment strategies. However, until date, few studies have investigated immune changes during MS pregnancy and with contradictory results.

In peripheral blood of MS patients, a shift to a Th2 response was observed during pregnancy, with a return to a Th1 profile after the delivery (10–12). Th17 frequency was examined in only one study with no significant changes (13). Regulatory T cells (Treg) were described to increase (14, 15), decrease (13), or not to be affected during gestation (16). CD56^bright^ NK cells were reported to increase during pregnancy with a decrease in the post-partum (16).

Decidual macrophages with an M2 phenotype and Treg are thought to be the key regulators of immune adaptations at the fetal–maternal interface (17). However, for MS patients no data are available on the local immune tolerance, where the most prominent changes occur.

Based on this ground, the aim of our study was to compare the immunomodulatory effects of pregnancy on five leukocyte populations (i.e., CD4^+^ and CD8^+^ T cells, CD4^+^CD127^−^CD25^high^ T cells [Treg], CD56^bright^CD16^−^ NK cells [NKbright], and CD14^+^CD163^+^ monocytes [M2]) in peripheral blood of MS patients and healthy women at different times during gestation. Moreover, for the first time, the same immune cell populations were also analyzed in decidual samples from the placenta of both MS and healthy women collected after delivery, to characterize maternal immunity at the placental interface.

## Methods

### Patients and Controls

Samples from healthy women (HC) and women with relapsing remitting (RR) MS were obtained from the Department of Surgical Sciences, Obstetrics and Gynecology, University of Torino, and from the Regional Reference Center for Multiple Sclerosis (CReSM), S. Luigi Gonzaga Hospital and Neuroscience Institute Cavalieri Ottolenghi (NICO), Orbassano.

Blood samples were collected from the following study cohorts: (I) 17 non-pregnant HC (G0); (II) 10 non-pregnant treatment-naïve RRMS patients (G0); (III) 8 HC at 8–10 weeks of gestation (I trimester); (IV) 11 RRMS patients at 8–10 weeks of gestation (I trimester); (V) 13 HC at 16–20 weeks of gestation (II trimester); (VI) 11 RRMS patients at 16–20 weeks of gestation (II trimester); (VII) 11 HC at 27–33 weeks of gestation (III trimester); (VIII) 11 RRMS patients at 27–33 weeks of gestation (III trimester); (IX) 9 HC in the 3 days after delivery (post-partum); (X) 6 RRMS patients in the three days after delivery (post-partum).

Decidual tissue were collected from 19 HC and 8 RRMS patients.

No patients experienced relapses during pregnancy. Treatment-naïve G0 patients did not suffer exacerbations or received corticosteroid during the last month before blood sampling.

The study was approved by the Ethic Committee of the University of Turin (June 18th, 2014). All the individuals gave their written informed consent.

The diagnosis of MS was performed according to the revised McDdonald's criteria (18). Clinical data were collected by neurologists at CReSM.

### Blood Samples Processing

Twenty-four ml of peripheral blood were collected in EDTA Vacutainers tubes (BD Biosciences, Milan, Italy), immediately kept in a refrigerated container at about 10°C and processed within 4 h after blood withdrawal. Peripheral blood mononuclear cells (PBMCs) were isolated by LymphoprepTM (Axis-Shiled, Olso, Norway) density gradient centrifugation. Cells were then cryopreserved at −80°C until use. After gentle thawing at 37°C, cells were immediately added to 5 mL RPMI 1640 (Invitrogen Life Technologies, Grand Island, NY, USA), supplemented with 10% heat-inactivated fetal bovine serum (FBS, Invitrogen Life Technologies) and centrifuged to remove DMSO (Sigma-Aldrich, St. Louis, MO). Cell pellets were re-suspended in RPMI 1640 medium supplemented with 10% heat-inactivated FBS and counted for flow cytometry experiments.

### Decidua Processing

Decidual samples were collected from HC and MS women after delivery at 38–40 weeks at the Department of Surgical Sciences, Obstetrics and Gynecology, University of Torino. Decidual and villous tissues were macroscopically identified and separated. Decidual tissue was washed, minced, and digested with 0.1 mg/mL collagenase IV and 0.01 mg/mL DNase I (Sigma-Aldrich) shaking in a water bath for 45 min at 37°C. Released cells were filtered through 100-, 70-, and 40-μm sieves (BD Labware) after a centrifugation with RPMI only (10 min at 1,500 rpm). Cells were re-suspended in RPMI 1640 medium supplemented with 10% heat-inactivated FBS and counted for flow cytometry experiments.

### Flow Cytometry

CD4^+^ and CD8^+^ T lymphocytes, Treg, NKbright and M2 monocytes were evaluated in blood specimen of HC and MS patients at different trimester of pregnancy. In addition, the same populations were analyzed in decidual tissues. The analyses were performed by a biologist blinded to the clinical data.

**Figure 1:**
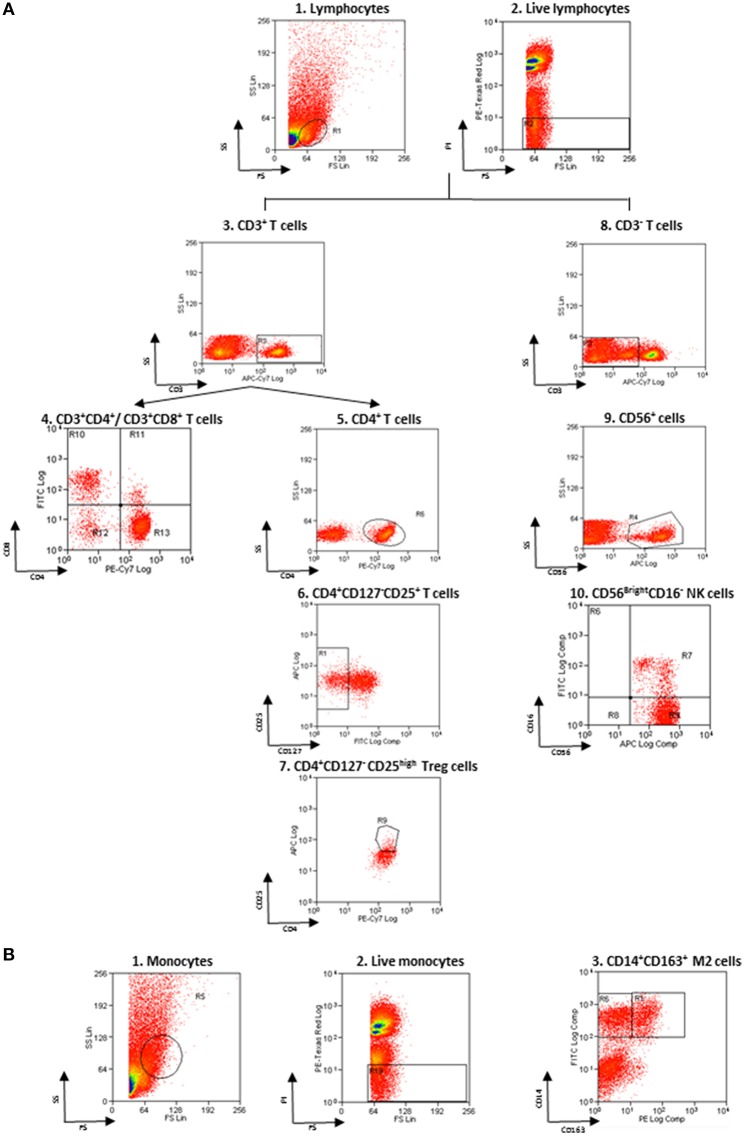
Flow cytometry gating strategy. **(A)** Gating strategy for lymphocytes and NK cells. (1) FSC vs. SSC gating was used to discriminate cells based on their size, (2) living cells were then identified by propidium iodide negativity. (3) CD3 was used as a T-cell marker. (4, 5) CD3^+^ cells were distinguished based on the presence of CD4 and CD8. (6, 7) CD127 and CD25 gating on CD3^+^CD4^+^ cells was used to select Treg. (8) CD3^−^ cells were gated based on the presence of CD56 and CD16 (9, 10) to identify NKbright. **(B)** Gating strategy for monocytes. (1) FSC vs. SSC gating was used to discriminate cells based on their size, (2) living cells were then identified by propidium iodide negativity. (3) CD14 and CD163 gating was used to identify M2 monocytes.

Non-specific sites of 3 × 10^6^ cells were blocked with rabbit immunoglobulins G (IgG, Sigma-Aldrich), and cells were then incubated with fluorochrome-conjugated monoclonal Ab (mAb) and isotype–matched negative controls for 10 min at 4°C. The following anti-human mAbs were used: CD163 Phycoerythrin (PE) and CD14 Fluorescein isothiocyanate (FITC) for M2 monocytes, CD3 allophycocyanin (APC)-Vio770, CD16 FITC and CD56 APC for NK cells, CD3 APC-Vio770, CD4 PE-Vio770 and CD8 FITC for T cells, CD4 PE-Vio770, CD25 APC, and CD127 FITC for Treg (Miltenyi Biotec, Bergisch Gladbach, Germany). Living cells identified by propidium iodide (Sigma-Aldrich) exclusion were gated according to their light-scatter properties to exclude cell debris. Samples were analyzed using a CyAn ADP, running Summit 4.3 software (Beckman Coulter, Brea, CA, USA). The gating strategy is shown in the Figure 1.

### Statistical Analysis

Statistical analyses were performed using R version 3.5.1 (www.r-project.org). Continuous data were presented as medians and ranges and categorical data were given as counts and percentages. The Shapiro-Wilk test was used to check for normality of distribution. The Kruskal-Wallis test with the Dunn's *post-hoc* test was used to compare continuous data between groups. Mann-Whitney *U*-test was used to compare cell percentages between different types of delivery in MS patients. *P*-values were adjusted for multiple comparisons using the Benjamini-Hochberg method to control the false discovery rate (FDR). Statistical significance was considered at *p* values <0.05.

## Results

### Demographical and Clinical Characteristics of MS Patients and HC

Demographical and clinical characteristics of individuals are summarized in Table 1. No significant differences were found between groups, with the exception of disease duration. As expected, disease duration was lower in non-pregnant MS patients compared to patients who donated blood samples in the post-partum or decidua samples (*p* = 0.04 and *p* = 0.02, respectively), since they are treatment-naïve and mostly newly diagnosed patients.

**Table 1:** Demographic and clinical characteristics of study cohorts.

	**Blood**										**Decidua**	
	**G0**		**I trimester**		**II trimester**		**III trimester**		**Post**			
	**HC (*n =* 17)**	**MS (*n =* 10)**	**HC (*n =* 8)**	**MS (*n =* 11)**	**HC (*n =* 13)**	**MS (*n =* 11)**	**HC (*n =* 11)**	**MS (*n =* 11)**	**HC (*n =* 9)**	**MS (*n =* 6)**	**HC (*n =* 19)**	**MS (*n =* 8)**
Age, years, median (interquartile range)^†^	28.53 (24–28.53)	28.5 (22.5–35.5)	34.5 (31.5–38.5)	34 (31–36)	34 (31–35)	33 (30–35.5)	33 (31–36.5)	33 (30.5–35.5)	35.33 (32–37)	33.5 (30.25–36.75)	34.5 (30.25–39.25)	33.5 (32.25–36.5)
Previous miscarriages, *n* (%)^*^			1 (20.0)	4 (44.4)	2 (22.2)	3 (30.0)	1 (12.5)	3 (30.0)	1 (11.1)	2 (33.3)	4 (22.2)	2 (25.0)
Caesarean section, *n* (%)											19 (100)	5 (62.5)
Disease duration, months, median (interquartile range)^§^		22.5 (11–46.75)		59 (46.75–73.50)		72 (53–90)		75 (56.5–90.5)		74 (63.25–88.50)		139.5 (107.5–169.8)
EDSS, median (interquartile range)^†^		0.5 (0–1.75)		1 (0.25–1.37)		1 (0–1.75)		1 (0–1.25)		1 (0.25–3.62)		1 (1–1.25)
Previous therapy, n (%)												
IFNβGlatiramer acetateNatalizumabNone				4 (36.4) 3 (27.3) 1 (9.1) 3 (27.3)		5 (45.4) 4 (36.4) 1 (9.1)		6 (54.5) 3 (27.3) 1 (9.1)		3 (50.0) 2 (33.3) 1 (16.6)		4 (50.0) 3 (37.5) 1 (12.5)
Months of wash out from therapy, median (interquartile range)^†^				1 (1–4.25)		4 (3–5)		7 (6–7)		7 (7–7.75)		8 (7.5–13.5)

† *Kruskal–Wallis test, p > 0.05*.

* *Total observations may change due to missing data*.

§ *Kruskal–Wallis test, p = 0.02 (Dunn's post-hoc test: G0 vs. post, p = 0.04; G0 vs. decidua, p = 0.02); MS, multiple sclerosis; HC, healthy controls; IFNβ, interferon beta*.

### Percentage of Immune Cell Populations in Blood and Decidua of MS Patients and HC

Each group of blood samples (G0, I, II and III trimester, post-partum) was analyzed for major immune cell subsets, in order to compare the effect of pregnancy on specific cell frequencies in women with MS and HC. Decidual tissues were analyzed for the same cell populations and percentages of decidual cells were compared with those from post-partum blood samples. Also, cell frequencies in decidual tissues from laboring and non-laboring delivery were compared.

### Adaptive Immune Cell Populations

**Figure 2:**
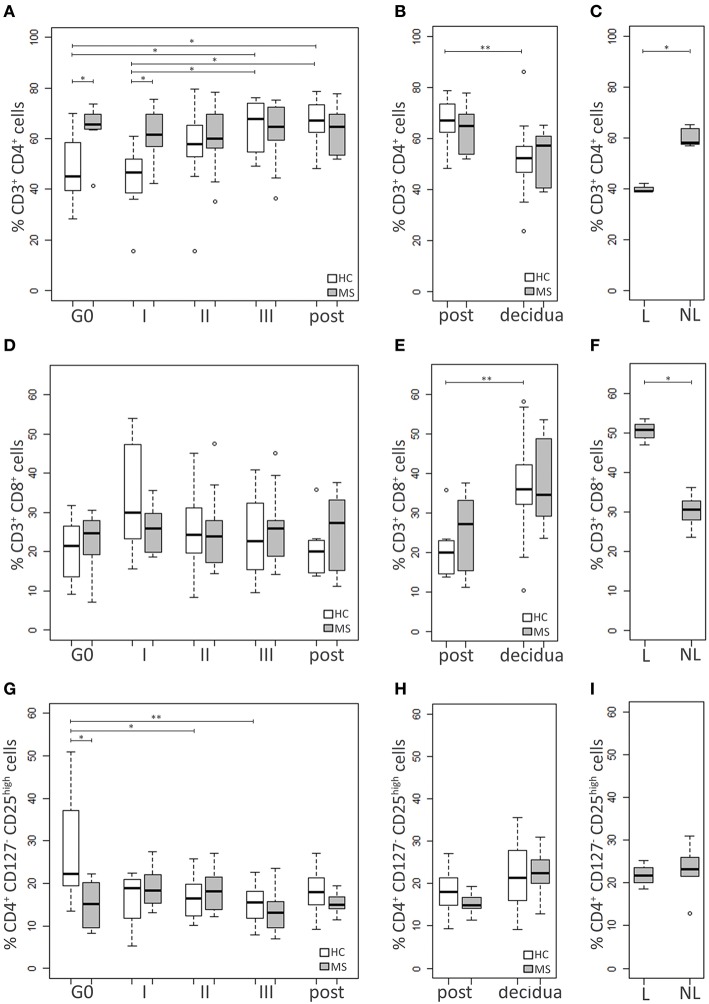
Adaptive immune cell populations. Comparison between blood cells of MS patients and HC at G0, I, II, and III trimester of pregnancy and post-partum: **(A)** CD4^+^ T cell percentage, **(D)** CD8^+^ T cell percentage, **(G)** Treg (CD4^+^CD127^−^CD25^high^) percentage. Comparison between cells in decidual tissues and post-partum blood samples of MS patients and HC: **(B)** CD4^+^ T cell percentage, **(E)** CD8^+^ T cell percentage, **(H)** Treg (CD4^+^CD127^−^CD25^high^) percentage. Comparison between cells in decidual tissues from laboring (L) and non-laboring (NL) MS patients: **(C)** CD4^+^ T cell percentage, **(F)** CD8^+^ T cell percentage, **(I)** Treg (CD4^+^CD127^−^CD25^high^) percentage. Kruskal-Wallis test with Dunn's *post hoc* test was used to compare cell percentages between MS and HC groups and between different time points. Mann-Whitney *U* test was used to compare cell percentages between different types of delivery in MS patients. *P* values were adjusted for multiple comparisons using the Benjamini-Hochberg method to control the FDR. ^*^0.01≤ *p* <0.05; ^**^0.001≤ *p* <0.01.

HC and MS samples were analyzed for three adaptive immune cell populations: CD4^+^ and CD8^+^ T cells and Treg (CD4^+^CD127^−^CD25^high^) (19) (Figure 2).

The percentage of CD4^+^ T cells was higher in the MS group compared to the HC at both G0 (*p* = 0.03) and I trimester of pregnancy (*p* = 0.03), while it was similar at the other time points. Notably, CD4^+^ T cell level increased during pregnancy only in the HC group, reaching the highest difference at the III trimester and the post-partum compared to both G0 (*p* = 0.03 and *p* = 0.02, respectively) and the I trimester (*p* = 0.02 and *p* = 0.01, respectively). On the contrary, MS patients showed similar levels at all time point analyzed (Figure 2A).

CD8^+^ T cell percentage did not differ between HC and MS women at G0 and remained relatively stable during pregnancy (Figure 2D).

The pre-pregnancy Treg level was lower in MS patients compared to HC (*p* = 0.008), while no significant differences between the two groups were found at the other time points. The Treg percentage of HC was found to be reduced during pregnancy, particularly at the II and the III trimester compared to G0 (*p* = 0.03 and *p* = 0.006, respectively). On the contrary, no significant changes over time were observed in MS patients (Figure 2G).

Decidual samples from HC and MS patients did not significantly differ for percentages of CD4^+^ T, CD8^+^ T and Treg (Figures 2B,**E**,**H**). The level of CD4^+^ T cells was higher in blood compared to the decidua, although the difference reached the statistical significance only in the HC group (*p* = 0.005; Figure 2B). On the contrary, the level of CD8^+^ T cells was higher in the decidua. This particularly applies to HC (*p* = 0.002), while in MS patients the difference was only approaching statistical significance (*p* = 0.06; Figure 2E). Treg percentages did not significantly differ between tissues, although in MS patients Treg seemed to be more frequent in the decidua compared to blood (*p* = 0.08; Figure 2H). Notably, in MS women with a laboring delivery, the level of CD4^+^ T cells was lower compared to MS women who had a cesarean section (*p* = 0.04; Figure 2C). The opposite behavior was observed for CD8^+^ T cells (*p* = 0.04; Figure 2F), while Treg levels were not influenced by the type of delivery (Figure 2I).

### Innate Immune Cell Populations

**Figure 3:**
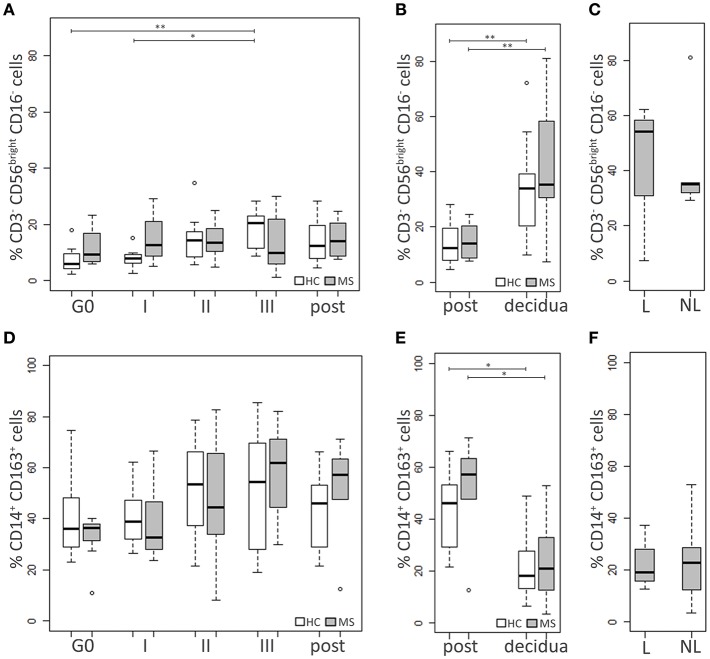
Innate immune cell populations. Comparison between blood cells of MS patients and HC at G0, I, II, and III trimester of pregnancy and post-partum: **(A)** NKbright (CD3^−^CD56^bright^CD16^−^) percentage, **(D)** M2 monocytes (CD14^+^CD163^+^) percentage. Comparison between cells in decidual tissues and post-partum blood samples of MS patients and HC: **(B)** NKbright (CD3^−^CD56^bright^CD16^−^) percentage, **(E)** M2 monocytes (CD14^+^CD163^+^) percentage. Comparison between cells in decidual tissues from laboring (L) and non-laboring (NL) MS patients: **(C)** NKbright (CD3^−^CD56^bright^CD16^−^) percentage, **(F)** M2 monocytes (CD14^+^CD163^+^) percentage. Kruskal-Wallis test with Dunn's *post hoc* test was used to compare cell percentages between MS and HC groups and between different time points. Mann-Whitney *U* test was used to compare cell percentages between different types of delivery in MS patients. *P* values were adjusted for multiple comparisons using the Benjamini-Hochberg method to control the FDR. ^*^0.01≤ *p* <0.05; ^**^0.001≤ *p* <0.01.

HC and MS samples were also analyzed for two cell populations of the innate immunity with immunoregulatory functions, namely NKbright (CD3^−^CD56^bright^CD16^−^) and M2 monocytes (CD14^+^CD163^+^) (Figure 3).

At each time point, no significant differences in the NKbright percentages were observed between MS patients and HC. In the HC group the level of the NKbright progressively increased during pregnancy, particularly at the III trimester compared to G0 (*p* = 0.006) and the I one (*p* = 0.03; Figure 3A).

The level of M2 cells did not significantly differ between HC and MS women. No statistically significant changes over time were highlighted in either group, although a modest increase of the M2 levels was observed at the III trimester and the post-partum compared to G0, particularly in MS patients (*p* = 0.12 and *p* = 0.20, respectively; Figure 3D).

Decidual samples from HC and MS patients did not significantly differ for the percentage of NKbright and M2 monocytes (Figures 3B,E). In both groups of individuals, the level of NKbright was significantly higher in the decidua compared to blood (HC: *p* = 0.006, MS: *p* = 0.009; Figure 3B), while higher percentages of M2 monocytes were found in blood compared to the decidua (HC: *p* = 0.01, MS: *p* = 0.01; Figure 3E). In MS patients, neither NKbright nor M2 monocyte levels were influenced by the type of delivery (Figures 3C,F).

## Discussion

Pregnancy represents the strongest known modulator of disease activity for patients with MS (4, 5). However, the mechanisms underlying this phenomenon are still unclear. Alterations in the frequency of specific immune cells involved in MS could be responsible of the disease amelioration. Here, we investigated immunological changes during pregnancy examining five different cell populations of both innate and adaptive immunity in blood of women with MS and HC at different times of gestation. The same cell subsets were also analyzed in decidual tissues of a cohort of MS patients and HC after the delivery.

As already stated, MS is thought to be primarily a CD4^+^ T cell-mediated disease, so it is not surprising that we observed higher levels of CD4^+^ T cells in peripheral blood of untreated non-pregnant MS patients compared to healthy non-pregnant women. Contrary to the HC group, in which CD4^+^ T cells increased since the II trimester of pregnancy, in MS patients the percentage of this cell type remained constant throughout the gestation and at high levels similar to those of non-pregnant patients. On the other hand, the frequency of CD8^+^ T cells did not differ between non-pregnant patients and controls and during gestation. All these findings are in agreement with those published by Airas et al. (16). CD4^+^ T cell population is highly heterogeneous, thus the stability of its total number in MS pregnancy may conceal changes in the balance among cell subsets, such as an increase in cells with an anti-inflammatory Th2 phenotype over pathogenic Th1 and Th17. Also, the proportion of cytotoxic CD4^+^CD28^null^ T cells, which have been recently associated to MS disease progression (20), could be affected by gestation thus contributing to disease amelioration. Likewise, the proportions of CD8^+^ T subtypes, such as naïve, effector and memory cells, could be altered throughout gestation. In pregnancy, CD8^+^ T cells are described as the most abundant T cell subset in decidual tissue, whereas CD4^+^ T cells are predominant in peripheral blood (21, 22). Accordingly, we observed lower levels of CD4^+^ T cells and higher levels of CD8^+^ T cells in the decidua compared to peripheral blood in both MS patients and HC. All HC decidual samples were collected from non-laboring cesarean sections, while women in the MS cohort had both cesarean or vaginal births. Notably, CD4^+^ and CD8^+^ T cell percentages were influenced by the type of delivery, as laboring decidua showed lower CD4^+^ and higher CD8^+^ T cell percentages compared to the non-laboring ones.

In addition to Th lymphocytes, CD4^+^ T cells also include T lymphocytes with regulatory properties. Treg are a group of heterogeneous cells, with functional and phenotypic distinctions. Due to the lack of agreement on the best markers for human Treg, there are conflicting reports in the literature regarding their definition, frequency and functions in both pregnancy and autoimmune diseases (23, 24). However, a growing body of evidence (25–30), as well as findings by our group (31, 32), suggest that these cells are numerically reduced and/or functionally impaired in MS patients. Accordingly, here we found decreased Treg levels in peripheral blood of untreated non-pregnant MS patients compared to non-pregnant HC. During pregnancy Treg levels declined in healthy women, while after delivery they did not significantly differ from non-pregnant women. On the other side, in MS patients Treg levels were not altered throughout gestation compared to both non-pregnant patients and post-partum. This is in agreement with Airas et al. (16) but in contrast with studies which describe an expansion (14, 15) or a decrease (13) of the Treg compartment during pregnancy in peripheral blood of MS patients. Notably, these reports make use of FOXP3 as a marker of Treg cells. Despite FOXP3 is an important determinant of Treg development and function, it is also highly expressed by the recently activated conventional T cells (33). Our observations are consistent with the hypothesis that pregnancy may alter the function of the Treg rather than their frequency, leading to an increased immunological tolerance. Furthermore, the systemic decrease of Treg may reflect their specific recruitment to the fetal-maternal interface, where they are required to suppress local immune response (34, 35). Indeed, we observed a moderate enrichment of CD4^+^CD127^−^CD25^high^ cells in decidual tissues.

Among the cellular components of decidual leucocytes, NK cells are reported to be the most abundant in physiological pregnancy (36). Unlikely NK in peripheral blood, which are mostly cytotoxic CD56^dim^CD16^+^ cells, they are predominantly CD56^bright^CD16^−^ cells with immunoregulatory functions (36). Accordingly, we observed higher levels of NKbright in decidual tissues compared to peripheral blood in both MS patients and healthy women. Circulating NKbright of MS patients have been reported to have similar frequency and phenotype compared to HC, but a significantly lower ability to inhibit T cell proliferation (37). Moreover, an expansion of these cells has been associated to a successful response to different MS-specific treatments (38) and to the disease remission observed during pregnancy (16). According to the literature, we did not find significant differences in the NKbright levels between non-pregnant patients and HC, but we observed an increase during pregnancy in HC, particularly in the III trimester (16). However, we did not detect such an increase in MS pregnancy. This is probably due to the different gating strategies adopted by Airas et al. who measured the frequency of all CD56^bright^ cells independently from their expression level of CD16. Notably, these results do not preclude that, while NKbright frequency was maintained at similar levels throughout pregnancy, their inhibitory activity was implemented.

Macrophages are the second largest type of immune cells in decidual tissue, accounting for ~20% of all the immune cells. During physiological pregnancy, most of the decidual macrophages exhibit properties predominantly associated with homeostatic M2 macrophages, suggesting a major role for these cells in the fetal immune tolerance (17). Circulating monocytes with an M2 commitment, such as CD14^+^CD163^+^ monocytes, preferentially differentiate into M2 macrophages in tissues (39). These monocytes/macrophages have been reported to accumulate in MS brains, in an attempt to switch off inflammation (40). Also, they have been demonstrated to induce the differentiation of Th2 and Treg cells in mice with experimental autoimmune encephalomyelitis treated with glatiramer acetate, thus ameliorating the disease course (41). As expected from these immature circulating cells, here we found a higher frequency of M2 monocytes in blood compared to the decidua in both MS patients and HC. These cells were not deficient in non-pregnant patients, but seemed to increase, although not significantly, in the third trimester and in the post-partum. This finding supports the role of monocytes/macrophages with an M2 phenotype as regulators of the immune system adaptation during pregnancy.

In conclusion, for the first time, we demonstrated that in MS patients the frequency of a large number of decidual immune cells at the time of delivery is comparable to that of HC. Although our analysis does not reflect the dynamic changes in the decidua that occur during the course of pregnancy, it describes the immune environment at the end of the third trimester, during which patients usually experience the greatest reduction in disease activity. Our findings suggest that a physiological immune regulation occurs at the fetal-maternal interface, regardless of pathological systemic processes. On the other hand, we observed some differences in the immunological changes induced by pregnancy in the peripheral circulation of patients and HC. In particular, the entire cell types analyzed seemed not to be modulated during MS gestation compared to non-pregnant patients, except for the M2 monocytes which showed a modest increase. Although these findings might have been influenced by inter-personal variability which affects cross-sectional studies, they reasonably support the hypothesis that MS pregnancy may result in a modulation of the immune cells functional activity rather than their frequency. Thus, further studies exploring functional changes of these cells would be crucial to bring light into the complex mechanisms of pregnancy-induced tolerance and autoimmunity overall.

## Data Availability

The datasets generated for this study are available on request to the corresponding author.

## Author Contributions

MS, LM, and ABe developed the hypothesis and designed the study. MS conceived the experiments and supervised the research. LM, DM, and EM collected placental samples and part of peripheral blood samples. GB processed placental samples to obtain deciduae. MS, FB, and ABa processed blood and decidual samples. SM, DM, and EM collected clinical data and organized the database. MS performed flow cytometry analyses. SM performed statistical analyses. MS and SM interpreted the data and wrote the first draft of the manuscript. SP and FM contributed to data interpretation and wrote sections of the manuscript. LM, ABe, and CB helped revised the manuscript.

### Conflict of Interest Statement

The authors declare that the research was conducted in the absence of any commercial or financial relationships that could be construed as a potential conflict of interest.
